# Dysregulation of genes coding for proteins involved in metabolic processes in mucopolysaccharidoses, evidenced by a transcriptomic approach

**DOI:** 10.1007/s11011-023-01231-5

**Published:** 2023-05-17

**Authors:** Karolina Pierzynowska, Patrycja Deresz, Grzegorz Węgrzyn, Lidia Gaffke

**Affiliations:** Department of Molecular Biology, Faculty of Biology, University of Gdansk, Wita Stwosza 59, 80-308 Gdansk, Poland

**Keywords:** Mucopolysaccharidoses, Transcriptomics, Regulation of metabolic processes

## Abstract

Mucopolysaccharidoses (MPS) are a group of lysosomal storage diseases (LSD) caused by mutations in genes coding for enzymes responsible for degradation of glycosaminoglycans (GAGs). Most types of these severe disorders are characterized by neuronopathic phenotypes. Although lysosomal accumulation of GAGs is the primary metabolic defect in MPS, secondary alterations in biochemical processes are considerable and influence the course of the disease. Early hypothesis suggested that these secondary changes might be due to lysosomal storage-mediated impairment of activities of other enzymes, and subsequent accumulation of various compounds in cells. However, recent studies indicated that expression of hundreds of genes is changed in MPS cells. Therefore, we asked whether metabolic effects observed in MPS are caused primarily by GAG-mediated inhibition of specific biochemical reactions or appear as results of dysregulation of expression of genes coding for proteins involved in metabolic processes. Transcriptomic analyses of 11 types of MPS (using RNA isolated from patient-derived fibroblasts), performed in this study, showed that a battery of the above mentioned genes is dysregulated in MPS cells. Some biochemical pathways might be especially affected by changes in expression of many genes, including GAG metabolism and sphingolipid metabolism which is especially interesting as secondary accumulation of various sphingolipids is one of the best known additional (while significantly enhancing neuropathological effects) metabolic defects in MPS. We conclude that severe metabolic disturbances, observed in MPS cells, can partially arise from changes in the expression of many genes coding for proteins involved in metabolic processes.

## Introduction

Mucopolysaccharidoses (MPS) are heterogeneous rare metabolic disorders from the group of lysosomal storage diseases (LSDs). They are caused by abnormalities in the function of specific hydrolases due to a genetic defect. Absence or deficiency of the activity of lysosomal enzymes, responsible for the process of degradation of glycosaminoglycans (GAGs), leads to their progressive accumulation (Zhou et al. 2020).

GAGs are long, linear polysaccharides formed from multiple repeating disaccharide subunits composed of an amino sugar and uronic acid or D-galactose. Once the chains are attached to the protein core, they can exist as proteoglycans. They are usually found on the cell surface or form an important part of the extracellular matrix, where they interact with the rest of the matrix. This provides a suitable environment for cell proliferation, differentiation and migration, and these compounds can interact with enzymes, structural proteins, transcription factors and growth factors or their receptors (Stapleton et al. 2018). In the degradation process, after the proteolytic removal of the preoteoglycan protein core, the released GAG chains are further cleaved and cut into fragments. In the case of a functional deficiency of one of the required enzymes, the process stops at a certain stage. Such partially degraded GAGs accumulate mainly intracellularly within the lysosome, thus disrupting its function and consequently affecting the function of the entire cell. Depending on which enzyme fails to perform its role, catabolism of heparan sulfate (HS), dermatan sulfate (DS), ketaran sulfate (KS), chondroitin sulfate (CS) or hyaluronate can be impaired (Kubaski et al. 2020).

Primary accumulation of the material, caused by impaired GAG degradation, is a pathogenic factor leading to disruption of cellular, tissue and organ homeostasis. This initiates the formation of numerous biochemical and structural secondary changes in patients' bodies, which contribute to causing disease symptoms. In addition to GAGs, cholesterol, phospholipids and glycosphingolipids can also be excessively accumulated as part of secondary storage (Heon-Roberts et al 2020).

There are 13 MPS types recognized to date, based on specific genes affected by mutations causing dysfunctions of corresponding enzymes responsible for GAG degradation (Wiśniewska et al. 2022). They are chronic, multisystem disorders and, over time, lead to a whole spectrum of symptoms associated with abnormalities in organ function; neurodegeneration occurs in majority of MPS types (Kobayashi 2019; Zhou et al. 2020). There can be a variety of clinical presentations, generally related to the type of GAG accumulated in the body. However, research is still needed to understand the details of the pathomechanisms behind the occurrence of individual symptoms. MPS show a progressive nature, thus, the patient's condition worsens with age, although the rate of progression itself is variable. Patients can vary in the severity of the disease, and the range of phenotypes includes cases of severe forms as well as debilitated ones with a picture that is difficult to diagnose early on (Wiśniewska et al. 2022). Life expectancy is shortened. Most often, in the absence of a therapy, people with MPS die prematurely (Muhlebach et al. 2011). Patients usually appear asymptomatic at birth; only later in early childhood do the first symptoms of the disease become apparent. Combined with the large number of possible clinical images, this fact poses a major problem in terms of detecting the disease (Wiśniewska et al. 2022).

Early presumptions on the mechanism of MPS suggested that GAG storage can be the only cause of the disease (Dorfman and Matalon 1976; Kelly 1976). Nevertheless, it appeared evident later that development of the disease depends also on secondary and tertiary changes in cells, including significant disturbances in metabolic processes (Gaffke et al. 2019; Fecarotta et al. 2020). Recently, it was demonstrated that expression of hundreds of genes is dysregulated in MPS cells which may significantly influence the cell physiology (Gaffke et al. 2020). In this light, a question appeared whether metabolic abnormalities, observed in MPS cells, arise solely from the primary GAG storage and resultant physical blockage of enzymes involved in various biochemical reactions or disturbed regulation of expression of genes coding for proteins involved in different metabolic processes can contribute significantly to the above mentioned abnormalities in the cellular metabolism. To answer this question, we have conducted a transcriptomic analysis, based on the results of studies on RNA isolated from fibroblasts derived from patients with 11 MPS types as well as control fibroblasts.

## Materials and methods

In this report, we have used the RNA-seq data, obtained by us previously (Gaffke et al. 2020) and deposited in the NCBI Sequence Read Archive (SRA) (accession no. PRJNA562649). In that work, fibroblasts derived from patients with following MPS types were used: I, II, IIIA, IIIB, IIIC, IIID, IVA, IVB, VI, VII, and IX. In control experiments, healthy human dermal fibroblasts (the HDFa line) were used. The detailed procedure has been described previously (Gaffke et al. 2020), and here we will provide the brief description, indicating the crucial parameters.

Illumina TruSeq Stranded mRNA Library Prep Kit was used to prepare mRNA libraries. After reverse transcription, cDNA libraries were sequenced with HiSeq4000 (Illumina, San Diego, California, USA) using the following parameters: PE150 (paired reads of 150 bp) and a minimum of 40 million raw reads, resulting in minimum 12 Gb of raw data per sample. Quality assessment was performed in FastQC version v0.11.7. The raw reads obtained were mapped to the human GRCh38 reference genome (from the Ensembl database) using Hisat2 version 2.1.0 software. Cuffquant and Cuffmerge software version 2 was used to calculate the expression levels of the transcripts along with the GTF file Homo_sapiens.GRCh38.94.gtf from the Ensembl database. The Cuffmerge program was run with the "library-norm-method classic-fpkm" setting, normalizing the expression values using the FPKM algorithm.

Statistical significance was assessed using one-way analysis of variance (ANOVA) for log_2_(1 + x) values with continuous normal distributions. The Benjamini–Hochberg method was used to prevent the problem of multiple comparisons (FDR; False Discovery Rate). Student's t-test with Bonferroni correction (*p* < 0.1) was used to compare the significance of changes between two groups. All statistical analyses were performed using R software version v.3.4.3. Annotation and classification of transcripts were performed using the BioMart interface to the Ensembl database.

## Results

**Fig. 1:**
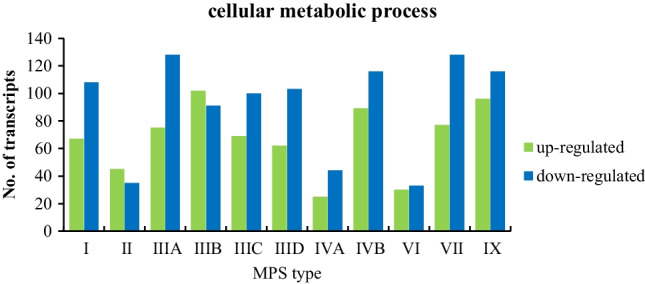
Number of transcripts coding for proteins involved in the cellular metabolic processes (GO:0044237) with changed levels of expression (at FDR < 0.1; *p* < 0.1) in different types of MPS relative to control cells (HDFa)

In order to determine abundance of changes in expression of genes coding for proteins involved in metabolic processes in MPS cells, we have analyzed the number of transcripts whose levels are significantly altered in MPS fibroblasts in comparison to healthy cells. These transcripts were extracted from the QuickGO database of the Gene Ontology Consortium (http://geneontology.org/) as the term “cellular metabolic process” (GO:0044237). The number of transcripts with altered levels (relative to controls) varied according to the type of MPS, nevertheless, it is clear that expression of many genes in this group was affected in each type of the disease (Fig. 1). The highest number of them (212) occurred in MPS IX, while the lowest (63) occurred in MPS VI. In each of the MPS types studied, we found both transcripts whose expression levels were significantly reduced and those where they were excessively high. However, it can be seen that in the vast majority of cases (except MPS II and MPS IIIB) there is a preponderance of decreased expression.

**Fig. 2:**
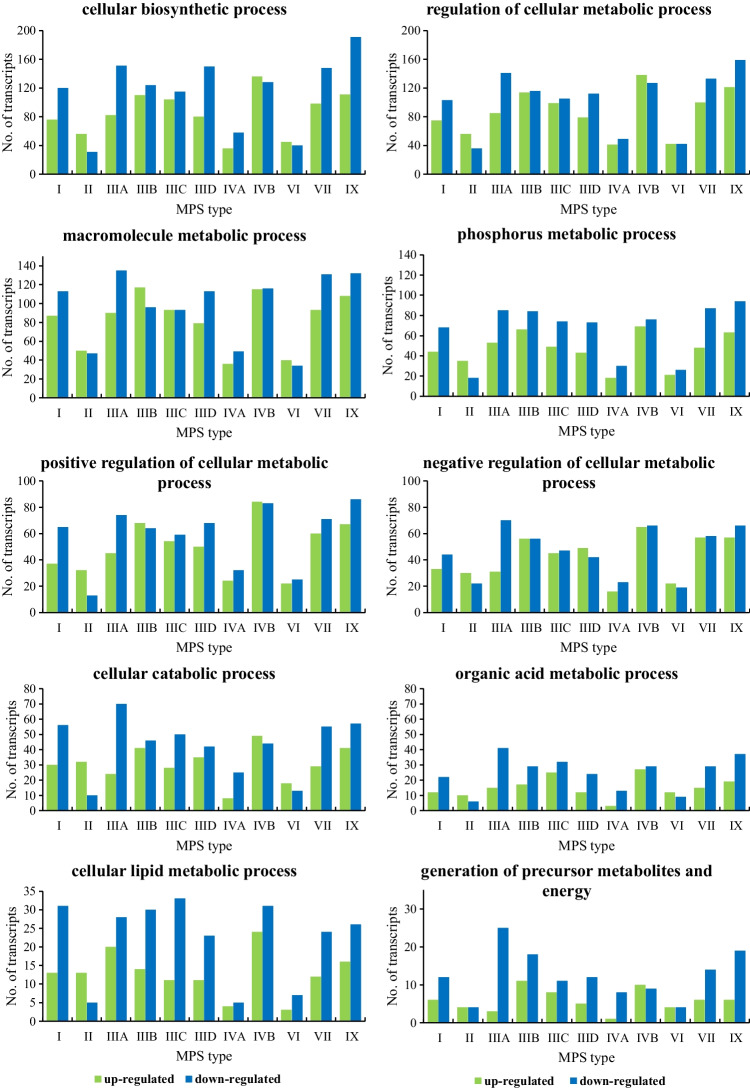
Number of transcripts, corresponding to genes from indicated sub-processes (child processes) of GO:0044237 (cellular metabolic process), defined according to the QuickGO database (cellular biosynthetic process (GO:0044249), regulation of cellular metabolic process (GO:0031323), macromolecule metabolic process (GO:0043170), phosphorus metabolic process (GO:0006793), positive regulation of cellular metabolic process (GO:0031325), negative regulation of cellular metabolic process (GO:0031324), cellular catabolic process (GO:0044248), organic acid metabolic process (GO:0006082), cellular lipid metabolic process (GO:0044255), and generation of precursor metabolites and energy (GO:0006091)), which levels were significantly changed in MPS cells relative to the control cells

To analyze in detail the differences in the expression of genes related to cellular metabolism in MPS, transcripts with altered expression levels were grouped into individual categories of subprocesses, according to the QuickGO database classification. The results are shown in Fig. 2. The selected subprocesses with the highest numbers of changes in gene expression included cellular biosynthetic process (GO:0044249), regulation of cellular metabolic process (GO:0031323), macromolecule metabolic process (GO:0043170), phosphorus metabolic process (GO:0006793), positive regulation of cellular metabolic process (GO:0031325), negative regulation of cellular metabolic process (GO:0031324), cellular catabolic process (GO:0044248), organic acid metabolic process (GO:0006082), cellular lipid metabolic process (GO:0044255), and generation of precursor metabolites and energy (GO:0006091).

**Fig. 3:**
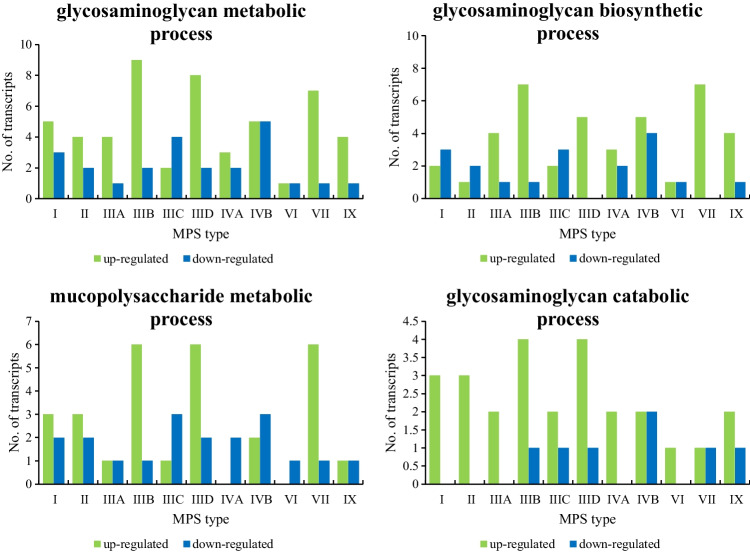
Number of transcripts coding for proteins involved in the metabolic processes of glycosaminoglycans, defined according to the QuickGO database (glycosaminoglycan metabolic process; GO:0030203), glycosaminoglycan biosynthetic process (GO:0006024), mucopolysaccharide metabolic process (GO:1903510), and glycosaminoglycan catabolic process (GO:0006027)) with changed levels of expression (at FDR < 0.1; *p* < 0.1) in different types of MPS relative to control cells (HDFa)

Changes in expression of genes coding for enzymes involved in GAG metabolism were of special interest in MPS cells. We found that several genes from this group were dysregulated in all investigated types of MPS (Fig. 3). Importantly, when considering genes whose products act in the process of GAG synthesis (glycosaminoglycan metabolic process; GO:0030203), enhanced expression of several of them was evident in most MPS types (Fig. 3). This might considerably influence the kinetics of accumulation of GAGs in MPS cells, as under conditions of impaired degradation of these compounds, any enhancement of their production should dramatically increase the storage level.

**Fig. 4:**
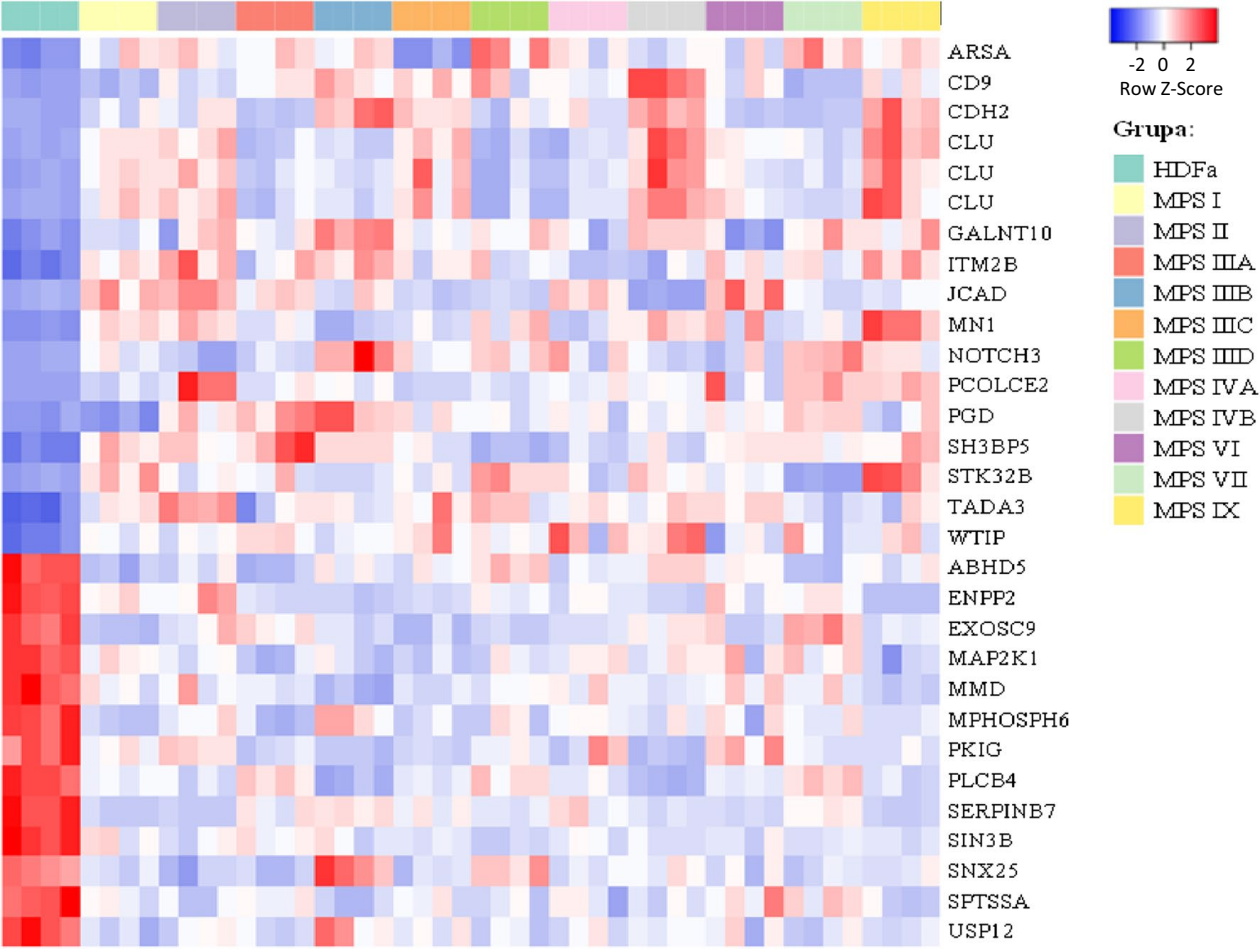
Heat map of transcripts coding for proteins involved in metabolic processes which expressions were significantly changed in at least eight MPS types relative to the control (HDFa) cells

To investigate whether there are transcripts common to most types of MPS with different expression levels from normal, we looked for transcripts levels were altered in at least 8 (out of 11 investigated) types of the disease. Thirty such cases related to cellular metabolic processes were found, as listed in Table 1, and the specific relations of the efficiency of gene expression are demonstrated in the form of the heat map in Fig. 4. Seventeen of transcripts (encompassing 15 genes, as 3 alternative transcripts of one gene, *CLU*, were identified) were those with increased expression (*ARSA, CD9, CDH2, CLU, GALNT10, ITM2B, JCAD, MN1, NOTCH3, PCOLCE2, PGD, SH3BP5, STK32B, TADA3, WTIP*), while the remaining thirteen (*ABHD5, ENPP2, EXOSC9, MAP2K1, MMD, MPHOSPH6, PKIG, PLCB4, SERPINB7, SIN3B, SNX25, SPTSSA, USP12*) were characterized by reduced expression.

**Table 1:**
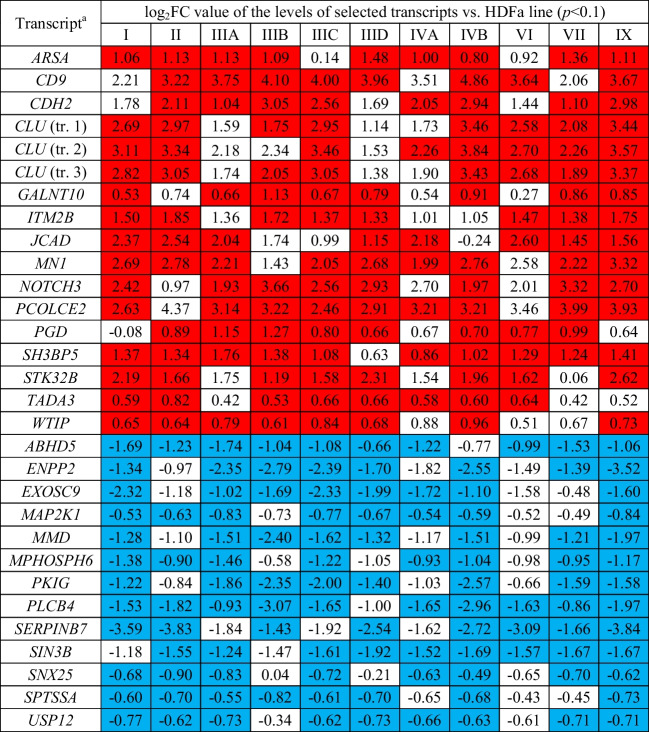
Transcripts with significantly changed levels in MPS fibroblasts relative to control (HDFa) cells (at *p* < 0.1) in at least 8 types of the disease

Up-regulation is marked in red, and down-regulation is marked in blue. Uncolored values indicate no statistically significant differences between MPS and HDFa cells

^a^ If more than one transcript of a specific gene was recognized, the relevant transcripts are marked as “tr. 1”, “tr. 2” etc.

In the next step, we looked for transcripts related to metabolic processes whose expression levels were particularly altered in different types of MPS. At least a 8-fold change (log_2_FC > 3 or log_2_FC < -3) in expression compared to cells of the control line (HDFa) was taken as the threshold for this analysis. The stated criterion was met by the 67 transcripts shown in Table 2. Interestingly, the highest number of transcripts whose expression has been so severely altered were in MPS IIIB (22 transcripts) and MPS IX (20 transcripts). In contrast, the least number of such cases was observed in MPS IVA (3 transcripts) and MPS VI (5 transcripts). Among the analyzed transcripts, those that met the accepted conditions (*p* < 0.1 and at least 8-fold change in the expression level) in at least 6 MPS types included: *CD9* (encoding the CD9 molecule that is a surface glycoprotein), *ID2* (encoding a protein that is an inhibitor of DNA binding), *OXTR* (encoding the oxytocin receptor), *PCOLCE2* (encoding an enhancer of procollagen C-endopeptidase), *PTGDS* (encoding prostaglandin D2 synthase), and *RPL23* (encoding a ribosomal protein that is a component of the 60S subunit). In addition, the most severely altered (as much as about 60-fold) expression levels were noted for the following transcripts: *OXTR* with increased expression in MPS IIIC and MPS IVB, *APOE* (encoding apolipoprotein E) with decreased expression in MPS IIIA, and *PTGDS* in MPS IIIA.

**Table 2:**
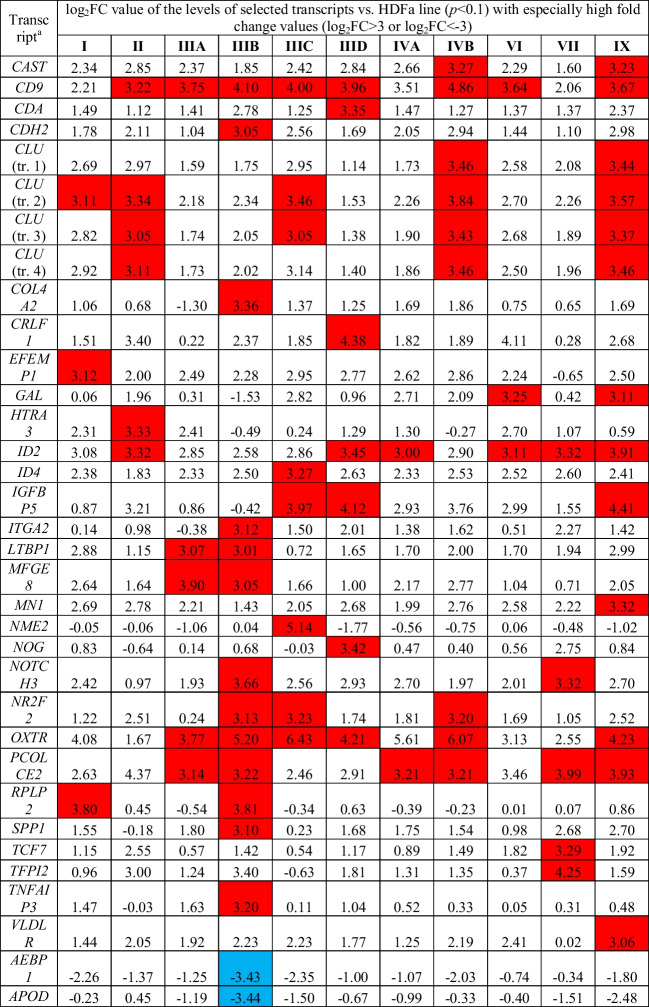

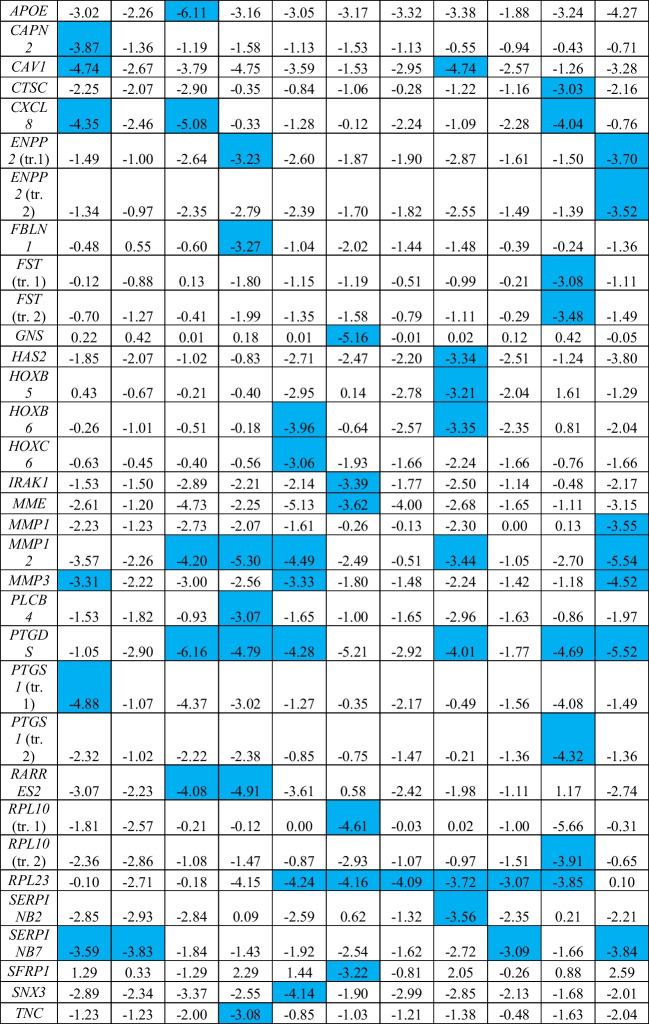
Genes coding for proteins involved in metabolic processes which expressions were significantly changed in at least eight MPS types relative to the control (HDFa) cells, with indication of log_2_FC

Colored boxes indicate statistically significant differences (at FDR < 0.1; *p* < 0.1) between MPS and control cell lines; blue boxes indicate down-regulation, and red boxes indicate up-regulation relative to the control

^a^ If more than one transcript of a specific gene was recognized, the relevant transcripts are marked as “tr. 1”, “tr. 2” etc.

**Fig. 5:**
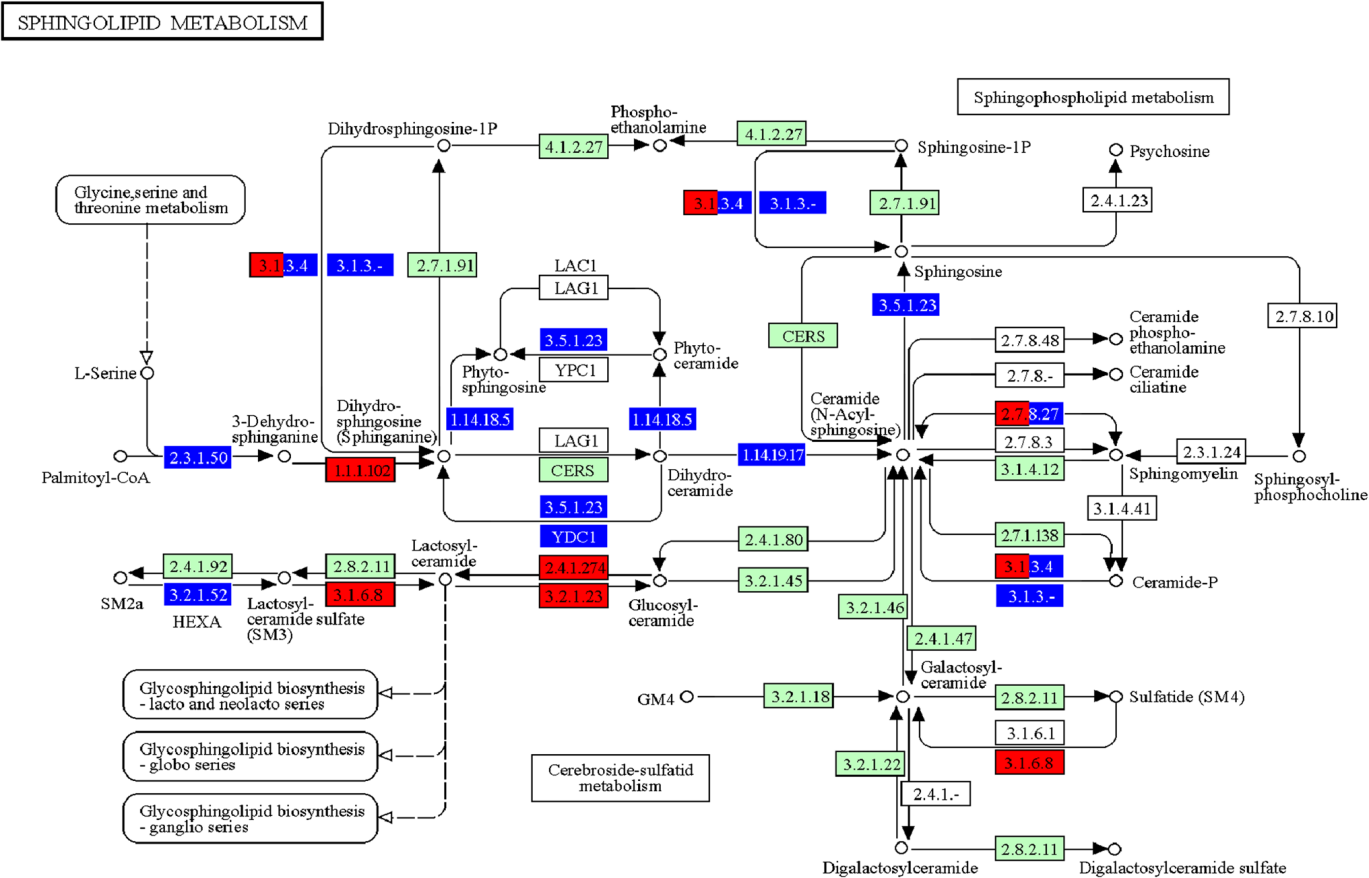
Changes in the sphingolipid metabolism in MPS cells as revealed by levels of expressions of genes coding for proteins involved in this process. The KEGG pathway presents the ‘sphingolipid meabolism’ process, imaged from transcriptomic data derived from MPS cells. Individual proteins or groups of proteins were colored if changes in the expression of indicated genes were observed in at least one type of MPS. Up-regulated and down-regulated genes (and corresponding gene products) are marked in red and blue, respectively. Green-marked boxes indicate results in which no statistically significant differences between MPS and HDFa were determined, and non-marked boxed show those with no transcriptomic results available (no expression in fibroblasts)

The group of the most severely accumulated secondary storage molecules in MPS cells which affect crucial functions, especially in the central nervous system, are sphingolipids and their derivatives (Anheuser et al. 2019; Saville and Fuller 2020). Our transcriptomic analysis with the use of the metabolic pathways’ visualization, employing the KEGG pathway database (https://www.genome.jp/kegg/pathway.html), indicated that many genes encoding proteins involved in the sphingolipid metabolism revealed changed expression in MPS cells (Fig. 5). This strongly suggest that enhanced expression of genes whose products are required for sphingolipid synthesis and/or impaired expression of those coding for sphingolipid degrading enzymes may significantly contribute to the secondary accumulation of these compounds in MPS cells.

## Discussion

MPS is a group of genetically determined LSDs with a progressive course (Nagpal et al. 2022). A mutation causing a defect in the activity of a specific lysosomal enzyme determines the onset of one of 13 types of the disease (Wiśniewska et al. 2022). The pathophysiology of MPS is undoubtedly related to the primary accumulation of particular type(s) of GAG(s) in patients' cells. However, it is now evident that this is a much more complex problem than previously thought. A large influence on the overall picture of the disease here is the presence of a number of secondary lesions that disrupt the normal course of cellular processes (Leal et al. 2022). However, we still do not understand all the molecular mechanisms underlying MPS, and recent years of research have shed light on many previously unknown aspects of MPS pathophysiology (Fecarotta et al. 2020; Leal et al. 2022; David et al. 2023; Pierzynowska et al. 2023). Along with these new discoveries comes the hope of developing alternative therapeutic approaches, as the commonly used current treatments do not deal with all the problems associated with MPS (Penon-Portmann et al. 2023).

Cellular metabolism is based on an interconnected, complex network of biochemical pathways and reactions that transform selected substrates to meet the energy needs of the cell. The products of these transformations are essential for the maintenance of normal cellular functions as well as the body as a whole. Maintaining homeostasis requires the concerted action of processes that are constantly involved in the degradation and synthesis of new molecules. In addition to being tightly regulated itself, metabolism is believed to have a signaling role. It allows cells to respond effectively to stimuli, including changes in substrate concentrations. Any disruption of this balance leads to pathological conditions and contributes to disease (Miyazawa and Aulehla 2018). Although MPS are, by definition, metabolic diseases, all possible abnormalities related to the regulation and course of these processes are still not fully understood. In fact, metabolomic studies reported the presence of severe metabolic abnormalities among patients with MPS III. These defects included most pathways related to amino acids, peptides, carbohydrates, lipids, nucleotides, vitamins and cofactors, energy formation or xenobiotic metabolism (Fu et al 2017; Tebani et al 2018).

The question remained whether global metabolic changes in MPS cells are caused by interactions between the primary storage material – GAG(s) – and other cellular compounds or by disturbed regulation of expression of many genes coding for proteins involved in various metabolic processes. The former possibility might be corroborated by recent discoveries that GAGs can physically interact with important proteins involved in cell signaling, like GPER1 and OXTR receptors (Pierzynowska et al. 2022). On the other hand, although until recently studies on global regulations of gene expression in MPS were scarce and focused on single types of the disease (Parente et al. 2012, 2016; Mazzoccoli et al. 2013; Salvalaio et al. 2017; Swaroop et al. 2018; Peck et al. 2019), subsequent transcriptomic analyses indicated that activities of hundreds of genes are affected in MPS cells which may cause perturbations in various cellular processes (Gaffke et al. 2020; Pierzynowska et al. 2020; Rintz et al. 2020; Brokowska et al. 2021; Cyske et al. 2022, 2023; Żabińska et al. 2023). In this light, we asked whether the latter hypothesis mentioned above, assuming the effects of changed expression of many genes on the cellular metabolism in MPS might be true.

The results of the analyses demonstrated in this report revealed the actual extent of the perturbation of regulation of expression of genes involved in metabolism-related processes in MPS. We found a significant number of transcripts whose products are involved in cellular metabolic processes with altered expression levels compared to the control fibroblasts (Fig. 1). Analysis of individual subprocesses extracted from the QuickGO database indicated the most impaired biochemical pathways which are related to the regulation of cellular metabolism and macromolecule metabolism (Fig. 2). Importantly, expression of genes involved in GAG metabolism was also significantly affected in MPS cells which may influence the course of the disease (Fig. 3). Especially, up-regulation of genes coding for enzymes required for GAG synthesis likely enhances the production of these compounds. It was demonstrated previously that a high efficiency of GAG production strongly correlates with a severe course of different MPS types (Piotrowska et al. 2009). Therefore, in cells with impaired GAG degradation, stimulation of their synthesis due to overexpression of specific genes can further enhance the storage and worsen the regulatory capacity in MPS cells, leading to a dangerous cascade of metabolic defects. Such a scenario might further explain the progressive course of the disease when the GAG storage level achieves the critical threshold.

Another important aspect of the MPS pathomechanism is the secondary storage of sphingolipids and their derivatives which significantly contribute to development of neurodegeneration and neuronopathic symptoms in MPS (Anheuser et al. 2019; Saville and Fuller 2020). It was proposed previously that such a secondary storage is caused by direct inhibition of sphingolipid activator proteins, including GM2 activator protein, saposin A, and saposin B (which are required for degradation of glycosphingolipids and gangliosides), by the primary storage compounds, like some GAGs (Breiden and Sandhoff 2020). On the other hand, our analysis using the KEGG pathways indicated that many genes coding for enzymes involved in the sphingolipid metabolism are dysregulated in MPS cells (Fig. 5). Therefore, the mechanism of the secondary storage of sphingolipids and related compounds in MPS may involve both direct inhibition of specific proteins by GAGs and impaired regulation of expression of genes coding for proteins responsible for balanced sphingolipid biochemical transactions.

Finally, we have identified a battery of genes which were dysregulated in most of tested MPS types (8 or more) (Table 1) and those whose expression is especially strongly changed in MPS fibroblasts relative to control cells (Table 2). Importantly, among all 30 transcripts identified to be significantly changed in most MPS types, every transcript was either up- or down-regulated in all MPS fibroblast lines (in other words, no transcript was up- regulated in some MPS types and down-regulated in others). This indicates that the investigated effects are common for all types of the disease. Among genes especially strongly affected in MPS cells, *CD9* and *CLU*, revealed particularly high overexpression. We suspect that increased levels of products of these genes may influence the course of MPS, as discussed below.

The *CD9* gene encodes a protein belonging to the tetraspanin family. It has a wide range of activity, being involved in cell adhesion, proliferation, signal transduction, sperm-oocyte fusion, regulation of inflammation, and tumor metastasis (Brosseau et al 2018). The CD9 protein physiologically interacts with integrins (Yu et al. 2017). This is an important feature with potential relevance in MPS types where there is an excessive accumulation of heparan sulfate. In such a case, this GAG may compete with CD9 for binding to the target receptor (Faye et al. 2009). Increased expression of this gene has also been shown in some brain tissues of a mouse model of MPS VII, where it is associated with late maturation of myelin (Parente et al. 2012).

Clusterin (also known as APOJ), is a multifunctional glycoprotein encoded by the *CLU* gene, and its different isoforms may be involved in distinct functions (Zhang et al. 2022). It participates in the process of apoptosis and is activated by stress conditions. In addition, it also acts as an extracellular chaperone protein, is associated with lipid transport and immune modulation. Elevated expression of the *CLU* gene is noted in refractory cancers, and is also associated with memory and cognitive impairment, as the structure of the brain may be altered under conditions of CLU dysregulation (Zhang et al. 2022). Specific variants of the *CLU* gene are strongly associated with an increased risk of Alzheimer's disease (Wilson and Zoubeidi 2017; Foster et al. 2019) and Parkinson's disease (Sampedro et al. 2020). Inhibition of *CLU* expression using siRNA was proposed as a therapeutic approach to treat these disorders (Wilson and Zoubeidi 2017). Symptoms similar to those occurring in the above mentioned diseases are also observed in neuronopathic types of MPS. Therefore, one might speculate that the widespread and strong upregulation of the *CLU* gene expression in MPS could contribute to the severity of these symptoms.

In conclusion, transcriptomic analyses presented in this report indicate that dysregulation of a battery of genes in MPS cells may significantly contribute to global metabolic disturbances observed in the group of mucopolysaccharide diseases. This includes, but is not restricted to metabolism of GAGs and formation of the secondary storage of sphingolipids and related molecules. Moreover, strongly enhanced expression of some genes, like *CD9* and *CLU*, may cause an independent enhancement of the metabolic burden, leading to elevated severity of symptoms, especially those related to neuropathology.

## Data Availability

RNA-seq data, deposited in the NCBI Sequence Read Archive (SRA), are available under accession number PRJNA562649.
